# Does Aging Have an Impact on Vitamin C Status and Requirements? A Scoping Review of Comparative Studies of Aging and Institutionalisation

**DOI:** 10.3390/nu15040915

**Published:** 2023-02-11

**Authors:** Anitra C. Carr, Masuma Zawari

**Affiliations:** Nutrition in Medicine Research Group, Department of Pathology and Biomedical Science, University of Otago, Christchurch 8011, New Zealand

**Keywords:** vitamin C, ascorbic acid, vitamin C requirements, vitamin C deficiency, ageing, aging, elderly, older, institutionalisation, institutionalised, NHANES

## Abstract

The global healthcare burden of an aging population continues to increase, with nearly a quarter of the total global burden of disease attributable to people aged ≥60 years. Older people are at greater risk of micronutrient deficiencies, including immune-supportive vitamin C, which is both a contributor to and a consequence of acute and chronic illnesses. However, whether healthy aging, per se, is associated with depleted vitamin C status and increased requirements for the vitamin is less certain. A systematic scoping review was carried out to assess comparative studies that reported the vitamin C status and prevalence of deficiency in older versus younger people and in older people relative to residential status. Furthermore, vitamin C requirements were assessed through comparative studies reporting vitamin C status and pharmacokinetics in older people relative to younger people. Overall, there was limited evidence to suggest that healthy aging, per se, is related to lower vitamin C status or higher requirements for the vitamin. However, institutionalised elderly had lower vitamin C status and enhanced vitamin C requirements, primarily as a result of low intakes and/or chronic illnesses, which were not being met by hospital or residential diets. Because institutionalised elderly are vulnerable to malnutrition and micronutrient deficiencies, it is imperative that appropriate nutritional interventions are instigated to provide optimal micronutrient intake to support healthy aging.

## 1. Introduction

The rapidly aging global population has imposed a growing burden on healthcare worldwide, with nearly a quarter of the total global burden of disease attributable to people aged ≥60 years [1,2]. Population aging is driving the worldwide epidemic of chronic diseases, with the leading contributors to disease burden in people aged ≥60 years being cardiovascular diseases, malignant neoplasms, chronic respiratory diseases, musculoskeletal diseases, and neurological and mental disorders [1]. The immune system is particularly weakened by advancing age, often referred to as immunosenescence, increasing the vulnerability to and severity of infectious diseases in older people [3]. This has become very apparent in the current coronavirus disease 2019 (COVID-19) pandemic, for which older people are at significantly higher risk of hospitalization and mortality [4]. Older people are also at greater risk for nutritional deficiencies due to physiological changes associated with aging, acute and chronic illnesses, medications, financial and social status, and functional decline [5].

Nutritional deficiencies in older people can be both a contributor to and a consequence of acute and chronic diseases, and this is particularly true for the immune-supportive nutrient vitamin C [6]. Vitamin C is an essential nutrient that cannot be synthesised by humans who require adequate and, due to its water-soluble nature, regular dietary intake of the vitamin to avoid hypovitaminosis C and potentially fatal deficiency. Daily intakes of 100–200 mg of vitamin C will provide adequate to saturating (50–70 µmol/L) plasma concentrations in healthy individuals, with concentrations ≤23 µmol/L indicating hypovitaminosis C and concentrations <11 µmol/L signifying deficiency and increased risk of developing scurvy [7]. Numerous studies have reported lower vitamin C status and a higher prevalence of deficiency in people with various acute and chronic conditions, including those most prevalent in older people [8,9,10]. Furthermore, higher vitamin C intake and circulating concentrations have been associated with healthy aging and decreased risk of morbidity and mortality [11,12]. These considerations are pertinent to global dietary intake recommendations [13]. To date, France is the only country that has a higher vitamin C intake recommendation category for older people, with a reference value of 120 mg/day for adults aged ≥75 years [14]. This was based on considerations related to supplemental vitamin C intake in older people as well as immunity, cardiovascular risk, cancer risk, and cognition [15].

However, whether vitamin C status and requirements are altered with healthy aging, i.e., in the absence of comorbidities, is less certain. The purpose of this comprehensive scoping review was to assess comparative studies reporting the vitamin C status and prevalence of deficiency in older versus younger people and in older people relative to residential status. Furthermore, vitamin C requirements were assessed through comparative studies reporting vitamin C pharmacokinetics in older versus younger people and observational studies reporting vitamin C status relative to intake stratified by age. Overall, healthy aging did not appear to be related to lower vitamin C status or higher requirements. In contrast, institutionalised elderly had lower vitamin C status and enhanced vitamin C requirements, primarily from lower dietary intakes and/or chronic illnesses, highlighting the vulnerability of institutionalised elderly. The additional requirements of institutionalised elderly can be met with increased intake of vitamin-C-rich foods or oral supplements.

## 2. Materials and Methods

A systematic scoping review of published literature was carried out using the National Library of Medicine PubMed database (updated 8 January 2023). Articles of interest included comparative studies of vitamin C status (and intake) in younger vs. older adults and in older free-living vs. institutionalised adults, as well as pharmacokinetic studies of vitamin C supplementation in older vs. younger adults. Boolean search strategies were used with the following title keywords: vitamin C, vitamins C, ascorbic acid, micronutrient*, antioxidant*, age, aged, ageing, aging, elderly, old, or older. Filters used for inclusion were human and English; filters used for exclusion were review, systematic review, clinical trial, randomised controlled trial, or meta-analysis. No limits were placed on the date of publication. Examples of search strategies: “vitamin C[Title] OR vitamins C[Title] OR ascorbic acid[Title]) AND (age[Title] OR aged[Title] OR ageing[Title] OR aging[Title] OR elderly[Title] OR older[Title] OR old[Title]) AND English[Filter] AND Humans[Filter] NOT (clinicaltrial[Filter] OR randomizedcontrolledtrial[Filter] OR review[Filter] OR systematicreview[Filter] OR meta-analysis[Filter]”, and “micronutrient *[Title] OR antioxidant *[Title]) AND (vitamin C[Title/Abstract] OR vitamins C[Title/Abstract] OR ascorbic acid[Title/Abstract]) AND (age[Title] OR aged[Title] ageing[Title] or aging[Title] OR elderly[Title] OR old [Title] OR older[Title]) AND English[Filter] AND Humans[Filter] NOT (clinicaltrial[Filter] OR randomizedcontrolledtrial[Filter] OR review[Filter] OR systematicreview[Filter] OR meta-analysis[Filter])”.

**Figure 1:**
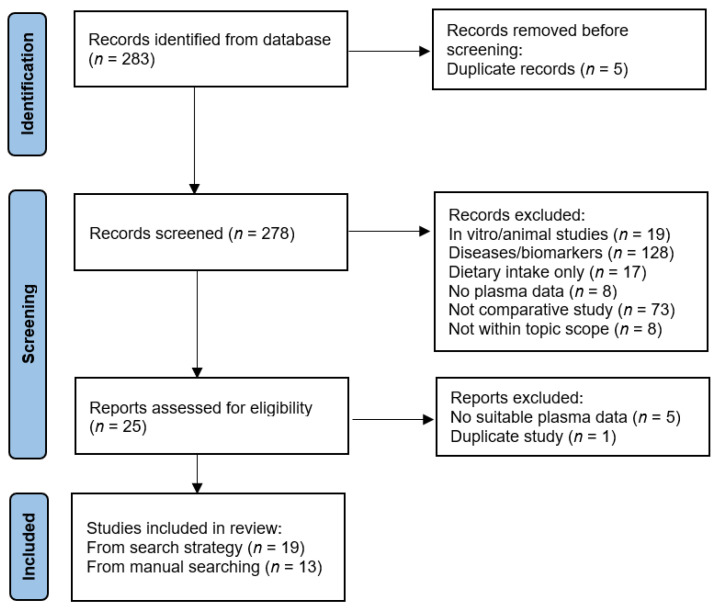
PRISMA flow diagram for study selection.

These search strategies resulted in 283 publication records that were imported into a spreadsheet for screening. Following removal of duplicates, titles and abstracts were screened and following removal of in vitro/animal studies, studies of specific diseases or related biomarkers, studies of intake only, studies with no plasma data, non-comparative studies, and out of scope studies, 25 records remained (Figure 1). Full texts were retrieved and a further 6 records were excluded due to insufficient or duplicate information, resulting in 19 publications. An additional 13 publications were identified through publication reference lists and manual database searches, totalling 32 comparative studies. The relevant data was extracted into tabular format and included: author and year, population and location of study, number and age of participants, vitamin C intake (if available) or dose administered (for pharmacokinetic studies), vitamin C status and/or prevalence of deficiency, and *p*-values. Vitamin C concentrations were reported as µmol/L, converted from mg/dL as required by multiplying by 56.8. The findings of these studies are summarised in the tables below with a narrative synthesis of the findings.

## 3. Results

The identified publications were categorised into age-related studies (*n* = 19) [16,17,18,19,20,21,22,23,24,25,26,27,28,29,30,31,32,33,34], requirements-related studies (*n* = 7) [22,26,35,36,37,38,39], and residence-related studies (*n* = 11) [23,27,33,34,40,41,42,43,44,45,46], with a number of studies spanning multiple categories [22,23,26,27,33,34].

### 3.1. Vitamin C Status Relative to Age

The relationships between aging and vitamin C status are shown in the comparative studies summarised in Table 1. In general, the non-institutionalised cohorts had adequate dietary vitamin C intakes of at least ~100 mg/day (higher if consuming supplements) and adequate circulating vitamin C concentrations of ~50 µmol/L, with higher values typically being observed in females, partly due to their lower body weight. Small studies carried out in the 1970s and 1980s suggested that older age may be associated with lower vitamin C status [24,26], although *p*-values were not reported in all cases [23,25]. Others have shown an inverse association between vitamin C status and ageing in men only [22] or even a small increase in vitamin C status in non-smoking older people [21], with the latter being attributed to higher supplemental intake in the older group. Large epidemiological studies have shown opposite trends to earlier studies, with an analysis of the US National Health and Nutrition Examination Surveys (NHANES 2003–2006 and 2017–2018) indicating higher vitamin C status and a lower or comparable prevalence of deficiency (<11 µmol/L) in people aged ≥60 [16,17,18]. The increased vitamin C status in older people appeared to be primarily related to higher vitamin C supplement intake [16]. Age categories <60 or <65 did not appear to be associated with differences in vitamin C status in the NHANES cohorts or the large French SU.VI.MAX study [16,17,18,19,20]. Studies carried out in hospitalised patients contrasted those carried out in the general population (Table 1), showing decreased vitamin C status (in the hypovitaminosis C range) and an increased prevalence of deficiency in older hospitalised patients [27,28]. The age effect, however, was less apparent after adjusting for disability, co-morbidities, smoking, and inflammation [28].

A number of studies have investigated the relationship of vitamin C relative to age in the old versus very old (Table 1), with two showing decreased vitamin C status and increased prevalence of deficiency in those aged ≥80 years [30,32] and one reporting a similar trend in men aged >75 years, but not women [31]. In contrast, three smaller studies in Germany, the UK, and the USA showed no significant trends in vitamin C status or prevalence of deficiency with increasing ages, >60 or >65 years [29,33,34].

### 3.2. Vitamin C Status Relative to Intake Stratified by Age

Relatively few studies have assessed vitamin C status relative to intake in different age groups (Table 2). Blanchard et al. [35,36,37] carried out detailed pharmacokinetic studies in healthy younger and older men and women. An initial steady-state study showed no differences in plasma vitamin C concentrations between younger and older women (aged 20–29 and 65–71 years, respectively) following three weeks of supplementation with 500 mg/day vitamin C [37]. In two follow-up studies carried out in both men and women [35,36], the participants were depleted via a month of a vitamin-C-restricted diet of <10 mg/day, followed by supplementation with a single dose of 500 mg vitamin C. There were no significant differences in the peak plasma concentrations of vitamin C between the two age groups. Similar findings were reported for participants who had first been repleted with three weeks of 500 mg/day vitamin C prior to the assessment of blood levels following a single 500 mg dose [35,36]. In contrast, Murata et al. [38,39] carried out comparative studies of long-term hospitalised older people (aged > 65 years) versus healthy young men and women (aged ≤ 40 years). Unsurprisingly, the older hospitalised patients had significantly lower baseline vitamin C status than the healthy young controls. Supplementation with a single dose of 250 mg of vitamin C did not alter their vitamin C status dramatically [39]; however, supplementation with 250 mg/d for 28 days did increase the participant’s vitamin C status, although the vitamin C status of the older people did not reach equivalent values to those of the younger people, suggesting higher utilisation of the vitamin in chronically ill older people [38].

Observational studies have reported associations between aging and higher vitamin C requirements [22,26]. Vitamin C intake versus plasma vitamin C concentrations stratified by age showed differences between younger and older men but not for women [22]. Specifically, at comparable vitamin C intakes, non-smoking men aged 60–88 years showed a lower response in their circulating vitamin C concentrations than non-smoking men aged 18–39 years [22]. An earlier study carried out on 34 nuns aged >65 and <65 years suggested that plasma vitamin C concentrations fell with advancing age, independent of changes in intake [26]. Multiple regression analysis of the data showed an overall decline of 3.4 µmol/L for every 10-year increase in age at a constant mean daily intake (*p* < 0.05).

### 3.3. Vitamin C Status Relative to Residential Status

A number of studies, including the UK National Diet and Nutrition Survey, have investigated the impact of institutionalisation on vitamin C status and the prevalence of deficiency (Table 3). In all cases, the participants who were institutionalised had dramatically lower vitamin C status and/or a higher prevalence of deficiency than non-institutionalised participants [23,33,40,41,42,43,45]; this was particularly apparent in older males in residential accommodation [44]. In the studies that reported dietary intakes, the institutionalised participants had much lower dietary intakes or a higher prevalence of inadequate intakes than the non-institutionalised participants [23,41,42,43]. Residents who were administered vitamin C supplements (50 mg/d) or who supplemented the institution diet with their own fresh fruit had higher plasma vitamin C concentrations than those who did not (*p* < 0.01) [44,45]. Several studies assessed the vitamin C status of hospitalised adults based on the place of residence prior to admission [27,34,46]. These also showed trends towards lower vitamin C status or a higher prevalence of deficiency in those admitted from institutions such as nursing homes.

## 4. Discussion

The comparative studies assessed in this scoping review indicate that healthy aging, per se, does not impact negatively on vitamin C status or requirements. The supplementation of depleted and repleted healthy young and older people showed no differences in the pharmacokinetics of vitamin C between the two groups [35,36]. Furthermore, epidemiological studies of the general population indicate that older people (aged ≥ 60 years) had a higher vitamin C status and a comparable or lower prevalence of deficiency than younger people [17,18]. The higher vitamin C status in older people was associated with intake of vitamin-C-containing supplements in the US cohorts [16]. Of note, there has been a gradual trend over time towards the increased usage of dietary supplements in the US, with adults aged ≥60 years being the largest multivitamin users [47]. Early comparative studies reported lower vitamin C status and a higher prevalence of deficiency in older people; however, these studies were also limited by small participant numbers and the use of older assay methods with low specificity [48]. Nevertheless, hospitalised older people appear to be more vulnerable to lower vitamin C status, likely a result of comorbidities and/or poor intake due to prior institutionalisation. Older people living in various institutions overwhelmingly have lower vitamin C status and a higher prevalence of deficiency than older people who are healthy and free-living; this is likely due to a combination of a higher prevalence of comorbidities and generally lower dietary intakes.

The lower dietary intake of vitamin C in institutions, such as nursing homes, has been found to be due to not only a lack of availability of vitamin-C-rich foods but also loss of vitamin C from the foods during preparation and delivery [42]. The supplementation of residents with vitamin C tablets (50–500 mg/day) or additional fresh fruit intake has been shown to overcome the lack of vitamin C in their diets and restore adequate vitamin C status [23,34,38,44,45]. Of note, following the discontinuation of supplementation, plasma vitamin C concentrations returned close to baseline levels within a week in long-stay residents [38]. This indicates that ongoing supplementation is required in institutionalised older people. Care also needs to be taken if supplying additional vitamin C through fresh fruit or vegetables due to their variable contents of the vitamin [8].

Few detailed investigations of vitamin C status relative to intake stratified by age have been carried out. Brubacher et al. [49] published a meta-analysis that included a subgroup investigation of the differences between adults (aged 15–65 years) and older people (aged 60–96 years) on plasma response to vitamin C intake. They reported a lower mean vitamin C status of older people (31 µmol/L) relative to adults (44 µmol/L) at an intake of 60 mg/day and concluded that the requirement for vitamin C is higher in older people. However, 33 of the 36 included studies were not directly comparative; thus, studies comprising different aged cohorts from various countries and from different eras were included, thereby precluding the direct comparison of the data. We identified seven comparative studies: two observational [22,26] and five interventional [35,36,37,38,39]. Of these, the most detailed pharmacokinetic studies were those of Blanchard [35,36,37]. They reported no significant differences in the pharmacokinetics of vitamin C administered to healthy younger and older volunteers.

In contrast, the studies of Murata et al. [38,39] compared chronically ill, institutionalised older adults with healthy young adults and showed lower vitamin C status in older people following long-term supplementation. The higher vitamin C requirements in chronically ill older people likely reflect enhanced disease-related demands for the vitamin, rather than aging per se [8]. It is noteworthy that hospital length of stay was found to be 2 days longer in people with hypovitaminosis C (*p* = 0.02), and these people also had fourfold higher odds of staying in hospital for >5 days relative to those with higher vitamin C status [27]. Although the micronutrient content of institution food has improved over time [50], it is still inadequate in many hospitals [51]. Due to the enhanced demands of acutely and chronically ill patients for vitamin C [8], especially during severe respiratory infections, to which older people are particularly susceptible [52], hospitalised older people would benefit from additional vitamin C supplementation.

A limitation of this scoping review was the inclusion of only studies that reported serum/plasma concentrations of vitamin C and not those studies reporting leukocyte or urinary concentrations of the vitamin. Plasma vitamin C has well-established thresholds for deficiency, hypovitaminosis C, adequacy, and saturation [53] and also correlates reasonably well with tissue status [54]. There can be issues, however, with using leukocytes and urine to assess vitamin C status. Some early studies reported differences in leukocyte vitamin C concentrations in institutionalised and non-institutionalised older people [55,56] and relative to young adults [23,37]. Although leukocyte vitamin C concentrations are believed to reflect tissue status, thresholds for deficiency and sufficiency are not well defined [57]. Moreover, leukocyte vitamin C concentrations do not necessarily reflect depleted states [58,59]. Urinary vitamin C values are an indirect measure of vitamin C status and require a test dose to estimate circulating status. Furthermore, lower excretion of vitamin C in older volunteers relative to young adults has been interpreted as signifying impaired gastrointestinal absorption [60], without consideration of initial body depletion status or potential enhanced requirements. Nevertheless, lower absorption of vitamin C could potentially be observed in older adults with inflammatory comorbidities due to the negative impact of inflammatory cytokines on intestinal vitamin C transport [61].

## 5. Conclusions

Overall, there is limited evidence to suggest that healthy aging, per se, results in lower vitamin C status or higher requirements for the vitamin. However, institutionalised older people have lower vitamin C status and enhanced vitamin C requirements due primarily to low dietary intakes and/or chronic illnesses; these additional demands can be met with increased intake of vitamin-C-rich foods or oral supplements. Because institutionalised older people are vulnerable to malnutrition and micronutrient deficiencies, it is imperative that appropriate nutritional interventions are instigated to provide optimal micronutrient intake to support healthy aging. Supplementation studies by Murata et al. [38] indicate that an additional 250 mg/day of vitamin C will help restore mean circulating concentrations close to adequate status in chronically ill, institutionalised older adults.

## Figures and Tables

**Table 1:** Vitamin C status relative to age.

Reference	Population (Location)	*n*	Age (Years)	Vitamin C Intake (mg/d)	Vitamin C Status (µmol/L)	Vitamin C Deficiency (%)	*p*-Value *^a^*
	**Younger adults vs. older adults**						
Powers 2023 [16]	4932 representative sampling of non-institutionalised population (NHANES, USA)	1463	20–39	NR	48 (45, 52)	7 (5, 9)	<0.001
		1563	40–59		49 (46, 53)	6 (4, 9)	
		1906	≥60		57 (53, 61)	7 (5, 10)	
Crook 2021 [17]	7607 representative sampling of non-institutionalised population (NHANES, USA)	2751	20–39	NR	NR	7 ± 1	
		2295	40–59			8 ± 1	
		2561	≥60			5 ± 1	<0.001
Schleicher 2009 [18]	4438 representative sampling of noninstitutionalised population (NHANES, USA)	725/815 *^b^*	20–39	NR	42/49 *^b^*	11/8 *^b^*	
		628/638	40–59		43/52	11/8	
		800/832	≥60		53/63	7/4	<0.001
Faure 2006 [19]	7074 people enrolled in the SU.VI.MAX study (France)	109/1798 *^b^*	35–45	99/95 *^b^*	53/59 *^b^*	NR	
		1006/1222	45–50	101/97	54/61		
		1375/1349	50–60	100/98	55/61		
		113/102	60–63	104/101	54/63		0.6
Galan 2005 [20]	3128 people enrolled in the SU.VI.MAX study (France)	NR	<45	NR	NR/60 *^b^*	NR	
			45–50		50/60		
			50–55		50/59		
			>55		51/62		0.6/0.9 *^b^*
Nakamura 2006 [21]	76 healthy non-smoking people (USA)	31	19–30	297 ± 278 *^c^*	31 ± 8	NR	
		45	59–86	565 ± 463	36 ± 12		0.02
Heseker 1994 [22]	2006 healthy people (Germany)	396/558 *^b^*	18–39	101/103 *^b^*	RC –0.21/–0.005 *^b^*	NR	0.001/0.4 *^b^*
		270/370	40–59	85/101			
		196/216	60–88	87/108			
Newton 1985 [23]	139 young and active older women (UK)	72	~36	NR	54 ± 22	NR	NR
		57	~73	58 (18–156)	44 ± 28		
Sasaki 1983 [24]	217 healthy people (Japan)	96/122 *^b^*	12–96	NR	r –0.453/–0.526 *^b^*	NR	<0.001
McClean 1976 [25]	80 non-smoking men (New Zealand)	22	17–29	NR	50	NR	NR
		4	30–39		64		
		17	40–49		35		
		24	50–59		47		
		13	60–69		40		
Burr 1974 [26]	34 nuns aged >65 and <65 years (UK)	22	19–63	NR	40	NR	
		12	69–89		24		<0.01
	**Hospitalised**						
Sharma 2019 [27]	147 general medical inpatients (Australia)	19	25–45	NR	NR	53 *^d^*	0.03
		28	45–65			75	
		100	>65			82	
Forster 2005 [28]	322 randomly selected hospitalised patients (UK)	129	<75	NR	21 (19, 36)	NR	
		193	≥75		15 (5, 33)		<0.01
	**Older adults vs. the very old**						
Jungert 2020 [29]	399 people aged ≥60 years (Germany)	399	60–96	CE 0.001	CE –0.075	NR	NS
Ravindran 2011 [30]	7228 randomly sampled clusters of rural and urban populations (India)	985/1080 *^c^*	60–64	NR	NR	69/37 *^e^*	
		658/864	65–69			72/39	
		552/575	70–74			81/42	
		287/275	75–79			79/45	
		186/176	≥80			85/51	<0.001
Birlouez-Aragon 2001 [31]	1987 people aged >60 years (France)	189/276 *^b^*	60–64	NR	↓/X *^b^*	↑/X *^b^*	0.02/0.2 *^b^*
		192/251	65–69				
		142/253	70–74				
		63/118	75–79				
		54/86	>80				
Mecocci 2000 [32]	107 community-dwelling people (Italy)	24	<60	NR	58 ± 3	NR	
		34	60–79		53 ± 3		
		17	80–99		42 ± 2		<0.01
		32	≥100		30 ± 2		<0.01
Barnes 1990 [33]	139 older people aged 60 to 90 years (USA)	54	60–70	NR	79 ± 32	NR	
		56	71–80		87 ± 37		
		29	81–90		79 ± 44		NS
Mandal 1987 [34]	277 older people newly admitted to assessment geriatric ward (UK)	58	65–74	NR	NR	40	NR
		143	75–84			40	
		76	≥85			41	

Data represent mean and SD or SEM, or median (Q1, Q3) or (95% CI). *^a^*—*p*-value is for vitamin C status (or % deficiency); *^b^*—male/female; *^c^* includes supplements; *^d^*—low levels (<28 µmol/L); and *^e^* North/South India. NR—not reported; NS—not significant; ↑—increase; ↓—decrease; X—no change; CE—coefficient estimate; RC—regression coefficient; NHANES—National Health and Nutrition Examination Survey; and SU.VI.MAX—Supplementation en Vitamines et Mineraux Antioxydants.

**Table 2:** Vitamin C status relative to intake stratified by age.

Reference	Population (Location)	*n*	Age (Years)	Vitamin C Dose (mg) and Time	Baseline Vitamin C (µmol/L)	Post-Suppl. Vitamin C (µmol/L)	*p* Value *^a^*
Blanchard 1990 [35]	30 healthy young and older men (USA)	15	25 ± 3	500 (4–5 h)	NR	53 ± 24	
		15	69 ± 3	500 (4–5 h)	NR	45 ± 16	NS
Blanchard 1990 [36]	28 healthy young and older women (USA)	14	26 ± 3	500 (4–5 h)	NR	56 ± 27	
		14	68 ± 3	500 (4–5 h)	NR	57 ± 24	NS
Blanchard 1989 [37]	16 healthy young and older women (USA)	8	20–29	500 (3 wk)	78 ± 24	93 ± 23	
		8	65–71	500 (3 wk)	93 ± 40	104 ± 15	NS
Murata 1995 [38]	40 long-term hospitalised older people and healthy young adults (Japan)	20	19–40	250 (28 d)	29 ± 9	59 ± 10	
		20	70–90	250 (28 d)	15 ± 6	47 ± 8	<0.01
Murata 1993 [39]	39 long-term hospitalised older people and healthy young adults (Japan)	20	19–35	250 (24 h)	31 ± 9	34 ± 9	
		19	66–96	250 (24 h)	17 ± 8	19 ± 7	<0.01

Data represent mean and SD. *^a^*—*p*-value is for young vs. old groups post-supplementation. NR—not reported; NS—not significant.

**Table 3:** Vitamin C status relative to residential status.

Reference	Population (Location)	*n* and Residential Status	Age (Years)	Vitamin C Intake (mg/d)	Vitamin C Status (µmol/L)	Vitamin C Deficiency (%)	*p* Value *^a^*
	**Institutionalised vs. community dwelling**						
Paniz 2007 [40]	67 older women (Brazil)	22 non-institutionalised	68 ± 6	NR	76 ± 6	NR	
		45 retirement home	71 ± 6		54 ± 4		0.002
Bates 1999 [41]	>1000 older people (NDNS, UK)	>785 free-living	≥65	33% *^b^*	44 ± 25	14	NR
		>230 institutionalised	≥65	45%	25 ± 21	40	
Löwik 1993 [42]	135 older women (the Netherlands)	52 independent living	≥65	146 ± 75	61 ± 21	0	
		29 serviced flats	≥65	135 ± 188	54 ± 19	4	
		54 nursing home	≥65	55 ± 28	24 ± 18	35	<0.001
Barnes 1990 [33]	139 older people (USA)	89 non-institutionalised	60–90	NR	89 ± 35	NR	
		50 institutionalised	60–90		70 ± 37		<0.01
Marazzi 1990 [43]	129 older women (Italy)	65 non-institutionalised	60–90	102 ± 68	59 ± 30	2	
		64 institutionalised	60–90	88 ± 42	37 ± 24	11	<0.001
Newton 1985 [23]	79 older women (UK)	57 active older people	~73	58 (18–156)	44 ± 28	NR	NR
		12 long-stay hospital	~81	14 (9–22)	13 ± 7		
		10 long-stay hospital	~88	26 (7–37)	10 ± 3		
Vir 1978 [44]	186 older people (Northern Ireland)	37 home	70–91	NR	22/30 *^c^*	30/23 *^c^*	NR
		43 hospital	65–94		16/27	30/14	
		20 sheltered dwelling	68–89		11/27	33/13	
		26 residential	65–95		10/23	67/24	
McClean 1977 [45]	70 older men (New Zealand)	35 living alone	NR	31 ± 29	26 ± 20	NR	
		35 residential home	>70	21	16 ± 15	83 *^d^*	<0.005
	**Hospitalised**						
Sharma 2019 [27]	149 general medical inpatients aged ≥18 years (Australia)	142 home	NR	NR	NR	75	
		61 home alone				80	
		7 nursing home				100	NS
Teixeira 2001 [46]	50 older patients admitted to internal medicine unit (France)	50 total cohort *^e^*	80 ± 9	NR	18 ± 18	88	
		5 nursing home	≥65		7 ± 1	NR	0.05
Mandal 1987 [34]	277 patients newly admitted to assessment geriatric ward (UK)	120 with family	≥65	NR	NR	43	NR
		94 living alone	≥65			33	
		20 sheltered residence	≥65			35	
		43 institutions	≥65			49	

Data represent mean and SD or SEM (or range). *^a^*—*p*-value is for vitamin C status (or % deficiency); *^b^*—<RNI (recommended nutrient intake); *^c^*—male/female; *^d^*—hypovitaminosis C (≤23 µmol/L); and *^e^*—total cohort comprised of 27 admitted directly from their homes, 14 from the emergency department, 5 from nursing homes, and 4 from other medical units. NDNS—National Diet and Nutrition Survey; NR—not reported; and NS—not significant.

## Data Availability

Not applicable.
